# Is air an issue? A severe case of subcutaneous emphysema in an immunocompromised patient

**DOI:** 10.1002/ccr3.5906

**Published:** 2022-05-23

**Authors:** Eloy F. Ruiz, David O. Rahni

**Affiliations:** ^1^ 12286 Department of Internal Medicine Rutgers New Jersey Medical School Newark New Jersey USA; ^2^ Department of Gastroenterology Cooperman Barnabas Medical Center RWJBarnabas Health Livingston New Jersey USA

**Keywords:** gas gangrene, gastrostomy, glioblastoma, subcutaneous emphysema, surgical wound dehiscence

## Abstract

Abdominal crepitus and air in the subcutaneous tissue should be recognized early, as the most common etiologies for subcutaneous emphysema are fatal if not treated acutely. We present the case of a patient who developed subcutaneous emphysema as a consequence of the dehiscence of a previously closed gastrocutaneous fistula.

## Graphical abstract

Recognize abdominal crepitus and air in the subcutaneous tissue as an entity that requires prompt and aggressive management; and identify key elements from the medical history and risk factors that could lead to its development.

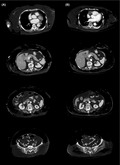

## 1 CASE PRESENTATION

A 67‐year‐old woman with a history of glioblastoma multiforme, receiving palliative bevacizumab and dexamethasone, presented with 1 week of abdominal distention. One month before admission, she underwent endoscopic clip placement for a gastrocutaneous fistula from a prior percutaneous endoscopic gastrostomy (PEG) tube.

**FIGURE 1:**
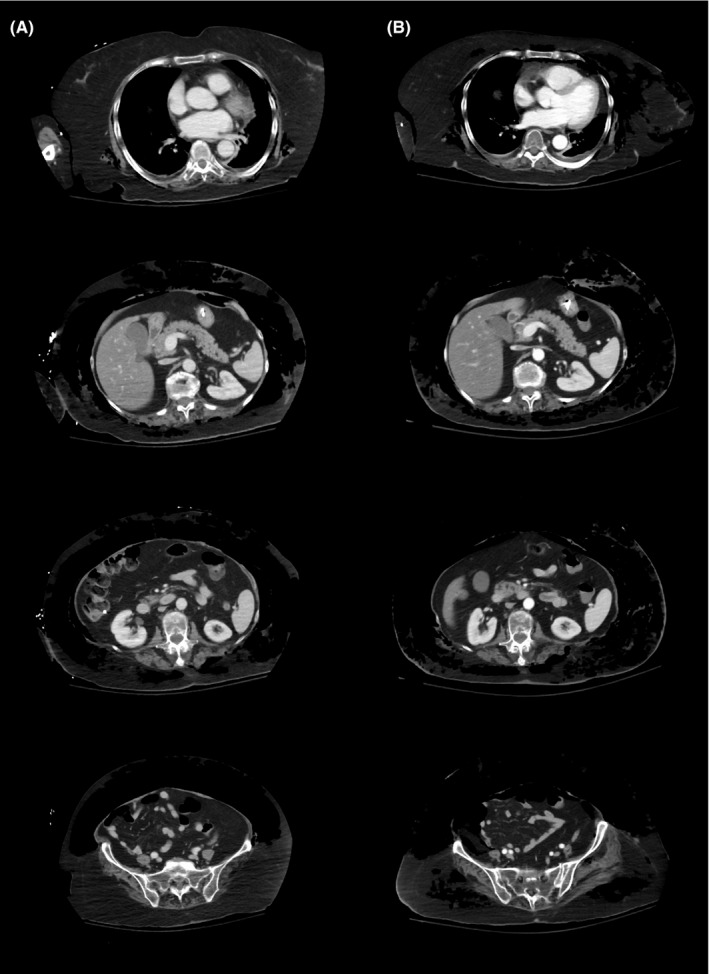
Initial (A) and follow‐up (B) computed tomography scans of the patient, showing worsening subcutaneous emphysema

Patient was afebrile and hemodynamically stable. An old PEG wound without purulence, erythema or bullae was observed. Crepitus was elicited on abdominal palpation without peritoneal signs. Laboratory tests were unremarkable. Urgent abdominal computed tomography showed extensive subcutaneous emphysema throughout the abdominal wall (Figure 1A) that progressively worsened and extended up to mid‐chest (Figure 1B). Pneumoperitoneum was not noticed.

Crepitus on examination should be recognized early as it warrants expedite work‐up for life‐threatening causes such as hollow viscus perforation, either traumatic or postoperative, with extension to subcutaneous fat, or infectious etiologies (gas gangrene). The subacute subcutaneous emphysema was secondary to the reopening of the gastrocutaneous fistula that was closed endoscopically 1 month before presentation. Risk factors for wound dehiscence in this case include obesity, terminal cancer and use of immunosuppressive agents.^1^, ^2^ Our patient was deemed at high risk for any surgical procedure, and hospice care was opted due to overall poor prognosis in a patient with underlying incurable cancer.

## AUTHOR CONTRIBUTIONS

All the authors (EFR and DOR) made substantial contribution (writing and edition) to the preparation of this manuscript and approved the final version for submission.

## CONFLICT OF INTEREST

The authors declared that they do not have a conflict of interest.

## ETHICAL APPROVAL

None.

## CONSENT

Written informed consent was obtained and signed by the patient and her family to publish this report in accordance with the journal's patient consent policy.

## Data Availability

None.
